# Optimum processing conditions for the maximum crystallization rate of poly(3-hydroxybutyrate-*co*-3-hydroxyhexanoate)

**DOI:** 10.1038/s41598-023-27595-3

**Published:** 2023-01-10

**Authors:** Khunanya Janchai, Takumitsu Kida, Masayuki Yamaguchi, Takenobu Sunagawa, Tetsuo Okura

**Affiliations:** 1 School of Materials Science, Japan Advanced Institute of Science and Technology, 1-1 Asahidai, Nomi, Ishikawa 923-1292 Japan; 2 Green Planet Technology Laboratories, Kaneka Corporation, 5-1-1 Torikainishi, Settu, Osaka 566-0072 Japan

**Keywords:** Green chemistry, Polymer chemistry, Engineering, Materials science, Environmental chemistry

## Abstract

The effect of thermal and shear histories on the crystallization rate of poly(3-hydroxybutyrate-*co*-3-hydroxyhexanoate) (PHBHHx) was studied. As with other crystalline polymers, the shear history greatly affected the crystallization rate when the shear rate was beyond a critical value, i.e., the inverse of the Rouse relaxation time. Even after the formation of extended chain crystals, spherulite texture was clearly discernable. It grew from certain points on the extended chain crystals. Consequently, a row of spherulites appeared along the flow direction. The resin temperature in the molten state was also significant. When the sample was heated to 170 °C, which is beyond the main melting peak in the differential scanning calorimetry curve, unmolten crystals did not affect the linear viscoelastic properties. They acted as effective nucleating agents for the rest of the polymer during cooling. Therefore, the shear history hardly affected the crystallization rate and the number of spherulites.

## Introduction

Plastic is a remarkable synthetic material because it is strong, durable, and lightweight, and can therefore be used as a more efficient substitute for other materials. The properties of a plastic can also be customized by altering the method of its synthesis and the additives included, making it useful in a wide range of industries and in our daily lives^1,2^. However, if not properly managed these wondrous materials will inevitably cause a waste crisis with disastrous consequences for living things and the environment. The solution to this problem necessitates proper waste management and the development of environmentally harmless plastics. Therefore, bioplastics and alternative materials with improved degradability have been invented^3–5^.

Poly(3-hydroxybutyrate) (PHB) is one of the most attractive bioplastics because it is produced from renewable resources and is easily biodegraded to carbon dioxide and water, even in the ocean^6–10^. However, it is prone to severe thermal degradation via the six-membered ring ester decomposition at the temperatures required for processing^11,12^. Although the degradation rate and its impact on processability have been predicted quantitatively, the poor processability of PHB cannot be ignored^13,14^. Therefore, an intensive study on the incorporation of another monomer species that would lower the melting point of the polymer was performed to enable low-temperature processing^15,16^. Poly(3-hydroxybutyrate-*co*-3-hydroxyhexanoate) (PHBHHx) is one such commercially successful copolymer. To date, PHBHHx has been used in various applications such as shopping bags, cutlery, straws, and food packaging^17,18^. To further extend the applicability of PHBHHx, it is necessary to increase its crystallization rate to reduce the cycle time and/or increase the production rate^19–23^. Therefore, considering actual processing operations, the crystallization behavior of a PHBHHx with various thermal and shear histories was examined in this study.

Up to now, there have been a number of studies on the crystallization behavior of PHB and its copolymers. According to them, PHB generally forms orthorhombic form, that is called α-form, under conventional cooling methods^24^, and its equilibrium melting point $T_{m}^{0}$ is around 200 °C^25^. Considering that the linear growth rate of crystallization becomes a maximum between $T_{m}^{0}$ and the glass transition temperature *T*_*g*_, which is around 10 °C^26^, the appropriate temperature to enhance the crystallization is around 105 °C^27^. This was supported by experimental results^28^. In general, PHB and its copolymers are known to form relatively large spherulites, because the nucleation process is quite slow. Therefore, various materials have been used to increase the nuclei, such as talc, boron nitride, hydroxyapatite, carbon nanotube, terbium oxide, uracil, thymine, orotic acid, benzoic acid^10^, behenamide and its derivatives, and diethyl 4,5,10,11-tetraoxo-3,6,9,12-tetraazatetradecane-1,14-dioate^29^. The flow-induced crystallization was also studied. It is well known that chain stretching during flow is responsible for the shish formation^21,30–34^. Therefore, a high-molecular-weight fraction that has a long characteristic time for chain stretching, i.e., Rouse relaxation time, plays an important role at the early stage of flow-induced crystallization, although PHB and its copolymers produced by microbes usually have narrow molecular weight distribution. Therefore, Fujita et al.^35^ added the high-molecular-weight PHB by solution mixing to induce the shish-formation and confirmed that the formation of shish-kebab structure was enhanced. However, in the industrial scale, it is not easy to add a small amount of a high-molecular-weight fraction homogeneously. The addition of a homopolymer to a copolymer is also often used to enhance the crystallization rate. In the case of PHBHHx, however, the thermal degradation becomes severe problem when using the homopolymer, i.e., PHB.

## Experimental procedure

### Material

The PHBHHx was manufactured by Kaneka Corp., Japan. The content of 3-hydroxyhexanoate is 5.4 mol%, and the melting point at the first heating of the sample pellets was 147 °C according to differential scanning calorimetry (DSC) measurements obtained at 10 °C min^−1^. The number-, weight-, z-, and z + 1-average molecular weights were *M*_*n*_ = 89,000, *M*_*w*_ = 179,000, *M*_*z*_ = 284,000, and *M*_*z*+1_ = 389,000 g/mol, respectively, as measured by gel permeation chromatography (GPC) using chloroform as a solvent and polystyrene as a standard. Multi-angle light scattering measurements combined with GPC were also used to evaluate the weight-average molecular weight. Based on the results, the Q factor was 0.457.

The PHBHHx pellets were dried for 3 h at 80 °C in a vacuum oven prior to the melting process. The obtained samples were molded into flat films (100–300 µm thick) using a compression molding machine (Table-type test press; Tester Sangyo Co., Ltd., Saitama, Japan) at 180 °C for 2 min. The films were cut into small pieces so that their rheological properties and crystallization behavior could be evaluated using a polarizing optical microscope (POM).

### Measurements

The frequency dependency of the oscillatory shear modulus was evaluated using a cone-and-plate rheometer (AR2000ex; TA Instruments, Inc., New Castle, DE, USA) at 150, 160, 170, and 180 °C under a nitrogen atmosphere. The diameter of the cone was 25 mm, the cone angle was 4°, and the frequency sweep was performed from 0.01 to 249.8 rad s^−1^.

The crystallization temperature was evaluated by DSC (DSC8500; PerkinElmer Inc., Waltham, MA, USA) under a nitrogen atmosphere. A film sample of approximately 3 mg was placed in an aluminum pan. After heating at 170 and 180 °C for 5 min, the sample was cooled at 10 °C min^−1^ to determine its crystallization temperature.

**Figure 1:**
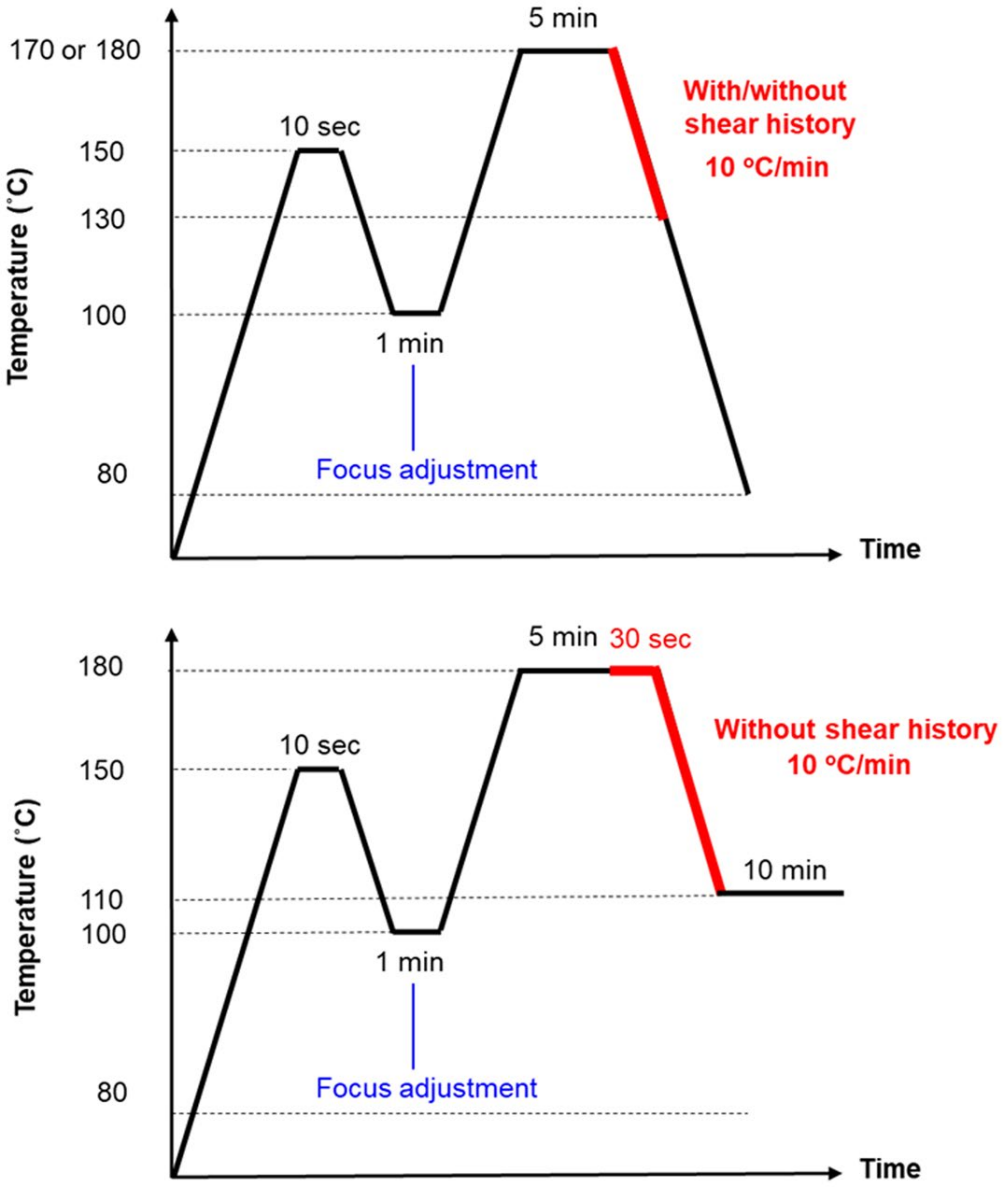
Experimental protocols. Investigations of crystallization during the cooling process (top) and the isothermal process (bottom).

The crystallization behavior of film samples with and without shear histories was also evaluated using a POM with crossed polar configuration (Leica DMLP; Leica Microsystems, Ltd., Wetzlar, Germany) attached to a parallel-plate shear stage made of quartz (CSS450; Linkam Scientific Instruments, Ltd., Surrey, United Kingdom). The parallel-plate stage was covered in a temperature-controlled chamber, which has a window 7.5 mm from the center that enables the passage of light. The angles between the flow direction and both the polarizer and analyzer were 45°. The gap between plates was 300 µm. The shear was applied during isothermal and cooling processes at 10 and 50 s^−1^ at the window position (7.5 mm from the center) by a rotation of the bottom plate. A photo-detector (PM16-121; Thorlabs Inc., Newton, MA, USA) was used instead of one of the eyepieces to determine the intensity of the light after it passed through a 633-nm color filter. A camera was set up on the other eyepiece to investigate the morphology. The details and some experimental results obtained by this machine were reported in our previous papers^36,37^. Figure 1 illustrates the experimental protocols for temperature and shear. The depolarized light intensity (DLI) value was calculated using the following equation:

1 $$DLI\;(\% ) = \frac{{I_{0} - I_{X} }}{{I_{//} - I_{X} }} \times 100,$$

where *I*_0_ is the light intensity passing through a sample under crossed polars, and *I*_*X*_ and *I*_*||*_ are the light intensities without a sample under crossed and parallel polars, respectively.

## Results and discussion

### Rheological properties

**Figure 2:**
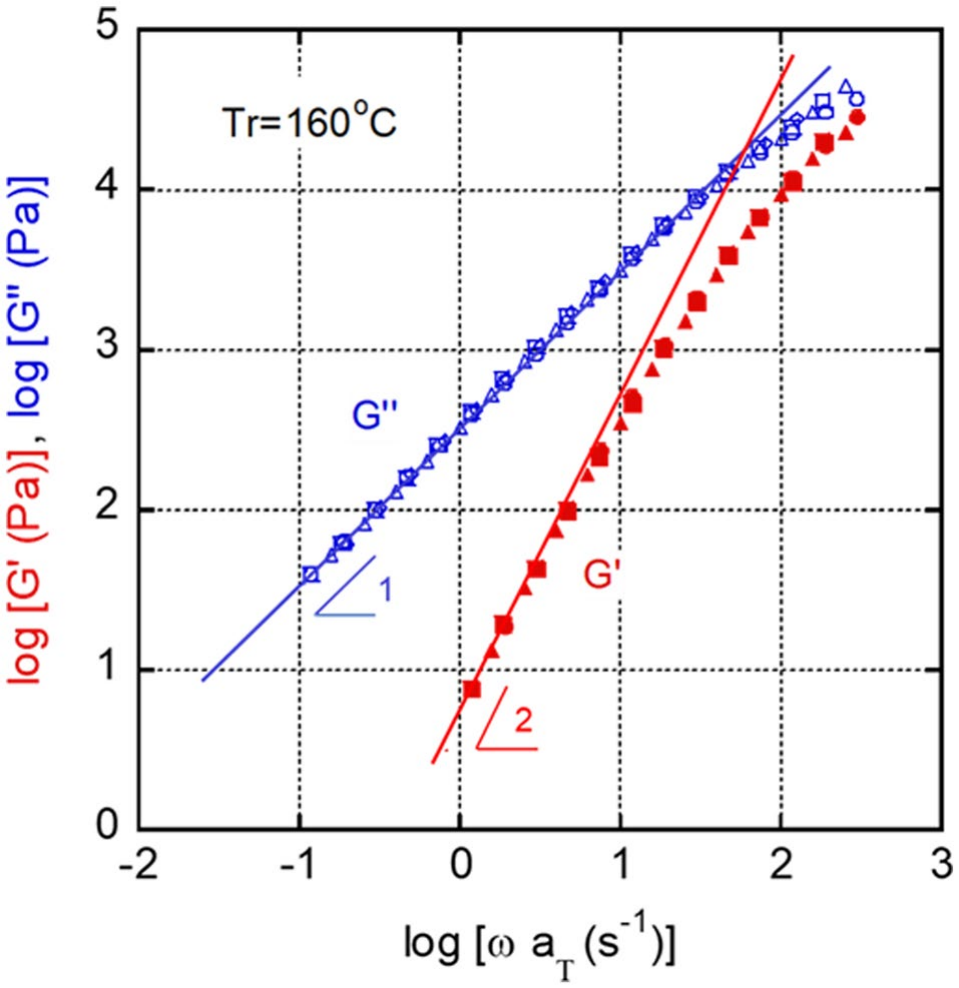
Master curves illustrating the frequency dependencies of the shear storage modulus *G*′ and the loss modulus *G*″. The reference temperature *T*_*r*_ was 160 °C.

The linear viscoelastic properties of the molten samples were evaluated before the measurements of crystallization, because rheological properties, which contain the information on molecular dynamics, directly affect the lateral growth rate of crystallization irrespective of the flow history. Figure 2 comprises the master curves illustrating the angular frequency *ω* dependence of the oscillatory shear moduli, i.e., the storage modulus *G*′ and the loss modulus *G*″, at 160 °C (the reference temperature *T*_*r*_).

Both *G*′ and *G*″ monotonically decreased with decreasing angular frequency. The slopes of *G*′ and *G*″ curves in the low-frequency region were 1 and 2, respectively, which are typical viscoelastic behaviors of a polymer melt^38^. Therefore, the rheological parameters in the terminal zone, i.e., the zero-shear viscosity $\eta_{0}$, steady-state shear compliance $J_{e}^{0}$, and weight-average relaxation time *τ*_*w*_, defined in Eqs. (2)–(4), were calculated to be $\eta_{0}$ = 3.2 × 10^2^ Pa s, $J_{e}^{0}$ = 4.0 × 10^–5^ Pa^−1^, and *τ*_*w*_ = 1.2 × 10^–2^ s at 160 °C.

2 $$\eta_{0} = \mathop {\lim }\limits_{\omega \to 0} \frac{{G^{\prime\prime}}}{\omega },$$

3 $$J_{e}^{0} = \mathop {\lim }\limits_{\omega \to 0} \frac{{G^{\prime}}}{{G^{{\prime\prime}2} }},$$

4 $$\tau_{w} \equiv \frac{{\int {\tau^{2} H\left( \tau \right)d\ln \tau } }}{{\int {\tau H\left( \tau \right)d\ln \tau } }} = \eta_{0} J_{e}^{0} ,$$

where *H*(*τ*) is the relaxation spectrum.

The *τ*_*w*_ value was also confirmed in Fig. 2. The inverse of the angular frequency at the cross point of the two straight lines was 1.3 × 10^–2^ s. As is well known, $J_{e}^{0}$ is strongly affected by the molecular weight distribution. According to Eq. (5), which was reported by Mills^39^, the $J_{e}^{0}$ value of monodispersed PHBHHx, i.e.,$J_{e}^{00}$, was calculated to be 7.2 × 10^–6^ Pa^−1^.

5 $$J_{e}^{0} = J_{e}^{00} \left( {\frac{{M_{z} }}{{M_{w} }}} \right)^{3.7} .$$

Using the relationship between $J_{e}^{00}$ and entanglement compliance $J_{N}^{0}$, the rubbery plateau modulus $G_{N}^{0}$, which can be estimated from Eq. (6)^40,41^, was calculated to be 3.5 × 10^5^ Pa. Previous researchers have evaluated the $G_{N}^{0}$ value for PHB and its copolymers. Liao et al.^42^ reported it to be 2.43–3.05 × 10^5^ Pa using PHBHHx samples with a 3-hydroxyhexanoate content of 3.8–10.0 mol%. Ebrahimi et al.^43^ evaluated a PHB homopolymer and found that its $G_{N}^{0}$ is 2.4 × 10^5^ Pa. According to Eq. (7), these data give the *M*_*e*_ values as 9400–14,500. Using the data obtained in the present study, $G_{N}^{0}$ was calculate to be 3.5 × 10^5^ Pa, which is slightly higher than the previously obtained values, presumably owing to experimental error in the molecular weight distribution. Only a tiny difference in the high molecular weight fraction, i.e., *M*_*z*_, produces a large difference in $J_{e}^{00}$. Moreover, $J_{e}^{0}$ may contain some experimental error.

6 $$G_{N}^{0} = \frac{1}{{J_{N}^{0} }} = \frac{2.5}{{J_{e}^{00} }},$$

7 $$M_{e} = \frac{\rho RT}{{G_{N}^{0} }}.$$

The horizontal shift factors *a*_*T*_ in Fig. 2 provided the flow activation energy *E*_*a*_ from the Arrhenius plot (Eq. (8)), which was calculated to be 36.9 kJ mol^−1^. The value corresponds to those reported previously^42,44,45^.

8 $$a_{T} = A\;\exp \left( { - \frac{{E_{a} }}{RT}} \right).$$

### Crystallization behaviors

**Figure 3:**
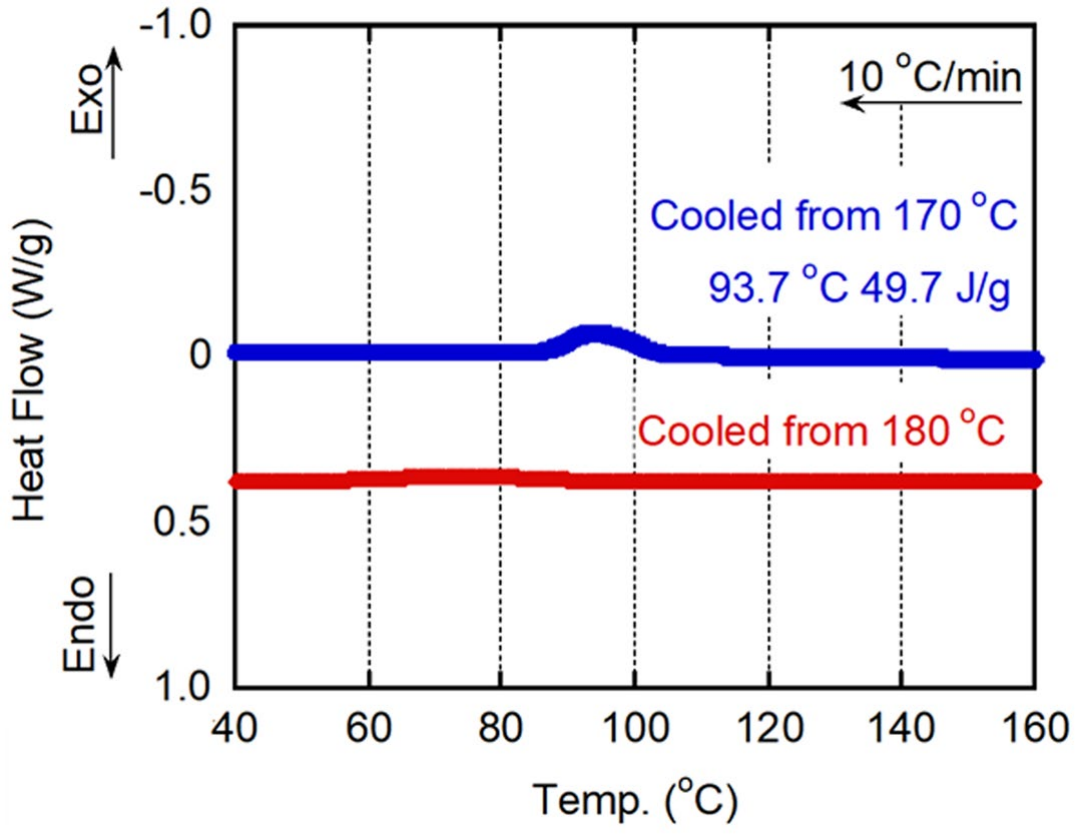
Differential scanning calorimetry (DSC) cooling curves obtained at 10 °C min^−1^. The samples were cooled from 170 and 180 °C.

Figure 3 comprises the DSC cooling curves obtained at a rate of 10 °C min^−1^. When the sample was cooled from 170 °C, an exothermic peak was clearly visible at 93.7 °C, with an onset temperature of approximately 103 °C. In contrast, a crystallization peak was not observed when the sample was cooled from 180 °C. The result suggested that crystal residues were fully melted once the sample was heated to 180 °C. In other words, a small number of crystals exist at 170 °C and act as nucleating agents for the molten material.

**Figure 4:**
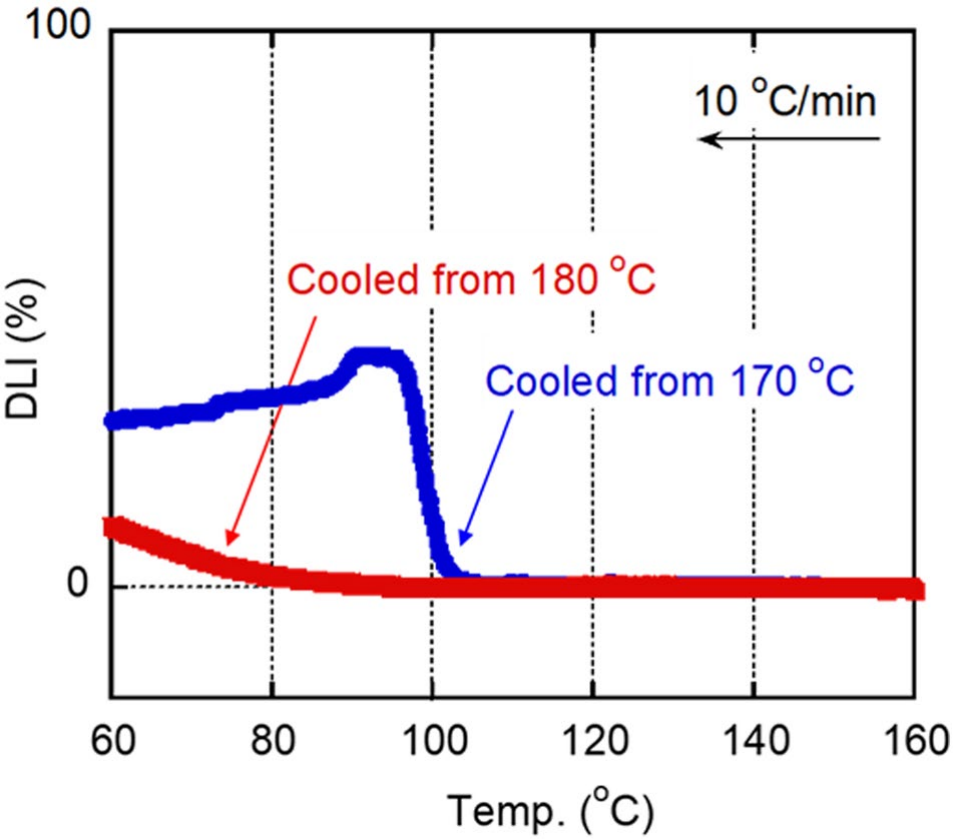
Growth curves of depolarized light intensity (DLI) during cooling at 10 °C min^−1^. The sample had no shear history before cooling.

The crystallization behavior was also investigated using a POM under the same conditions used for the DSC analysis. Figure 4 comprises the depolarized light intensity (DLI) curves obtained during cooling from 170 and 180 °C. The DLI value started increasing at approximately 103 °C when the sample was cooled from 170 °C, which corresponded well with the DSC result. However, when the sample was cooled from 180 °C, the value increased slightly at 75 °C. Considering that the DSC curve did not feature any exothermic peak/shoulder, even at 75 °C, the POM observation seems to be more sensitive in the early stage of crystallization. The decrease in the DLI values after passing the maximum in the cooling curve from 170 °C must be attributed to the increase in the light scattering from spherulites.

**Figure 5:**
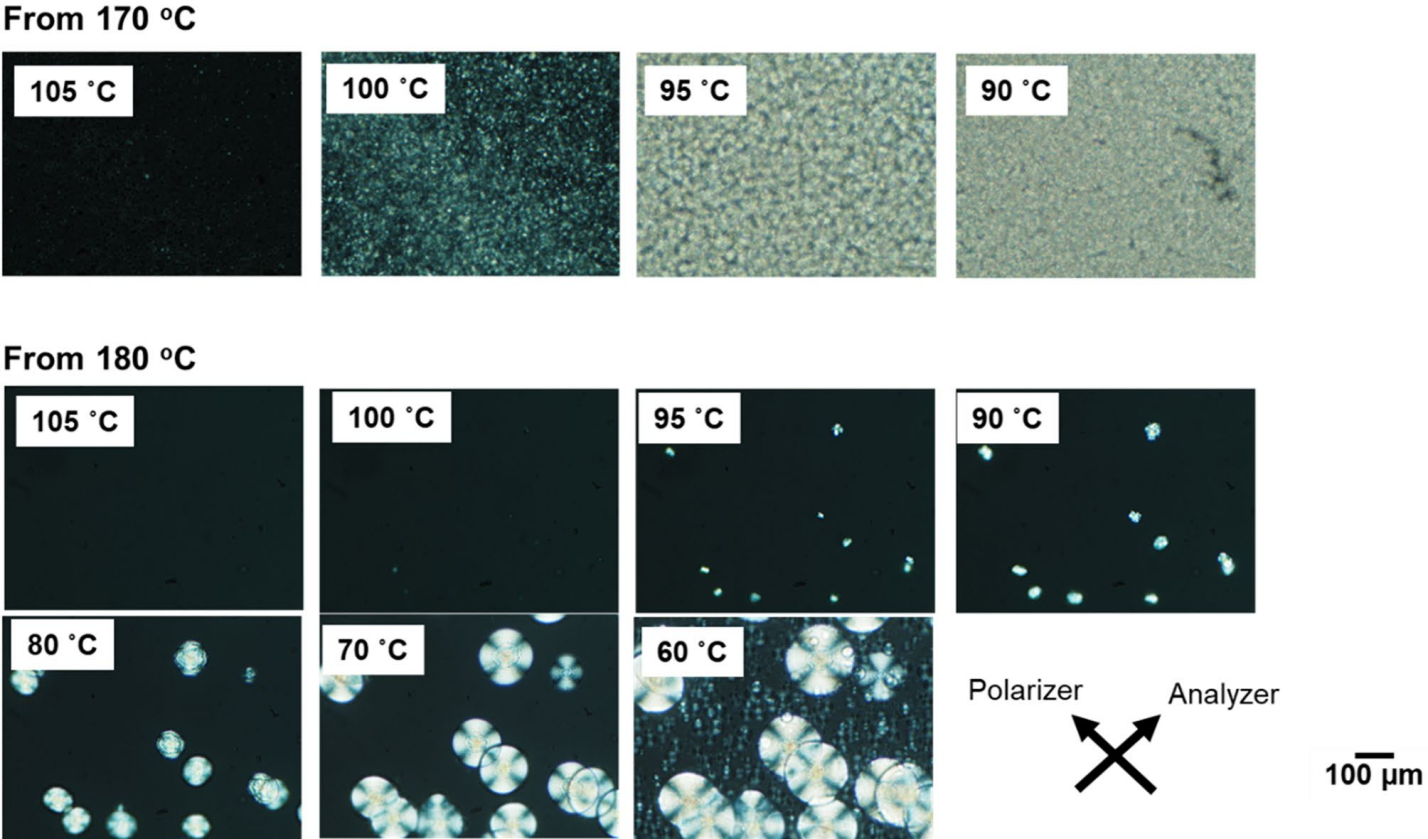
Polarizing optical microscope (POM) images obtained using crossed polars that indicate the morphology of the samples during cooling from 170 °C (top) and 180 °C (bottom) without shear history. The cooling rate was 10 °C min^−1^.

The morphological information obtained during cooling is shown in Fig. 5. It should be noted that the POM images do not always correspond with the DLI value because the shutter speed of the camera was selected automatically to capture the image clearly. Therefore, when the DLI was low, a slow shutter speed was selected to avoid a dark image.

Irrespective of the heating temperature before cooling, i.e., 170 or 180 °C, spherulite texture was detected without any macroscopic orientation. However, the number of spherulites differed significantly. Numerous spherulites were detected when the sample was cooled from 170 °C. This is as expected because at 170 °C the unmolten crystals acted as nuclei. Moreover, it should be noted that these unmolten crystals hardly affected the linear viscoelastic properties because the rheological terminal region was clearly detected, even at 150 °C, as shown in Fig. 2. This means that crystals existed as solid particles having no interaction with polymer chains in the molten state. Once the crystals provide fringed micelle structure and/or network structure, a long-time relaxation mechanism must be detected.

**Figure 6:**
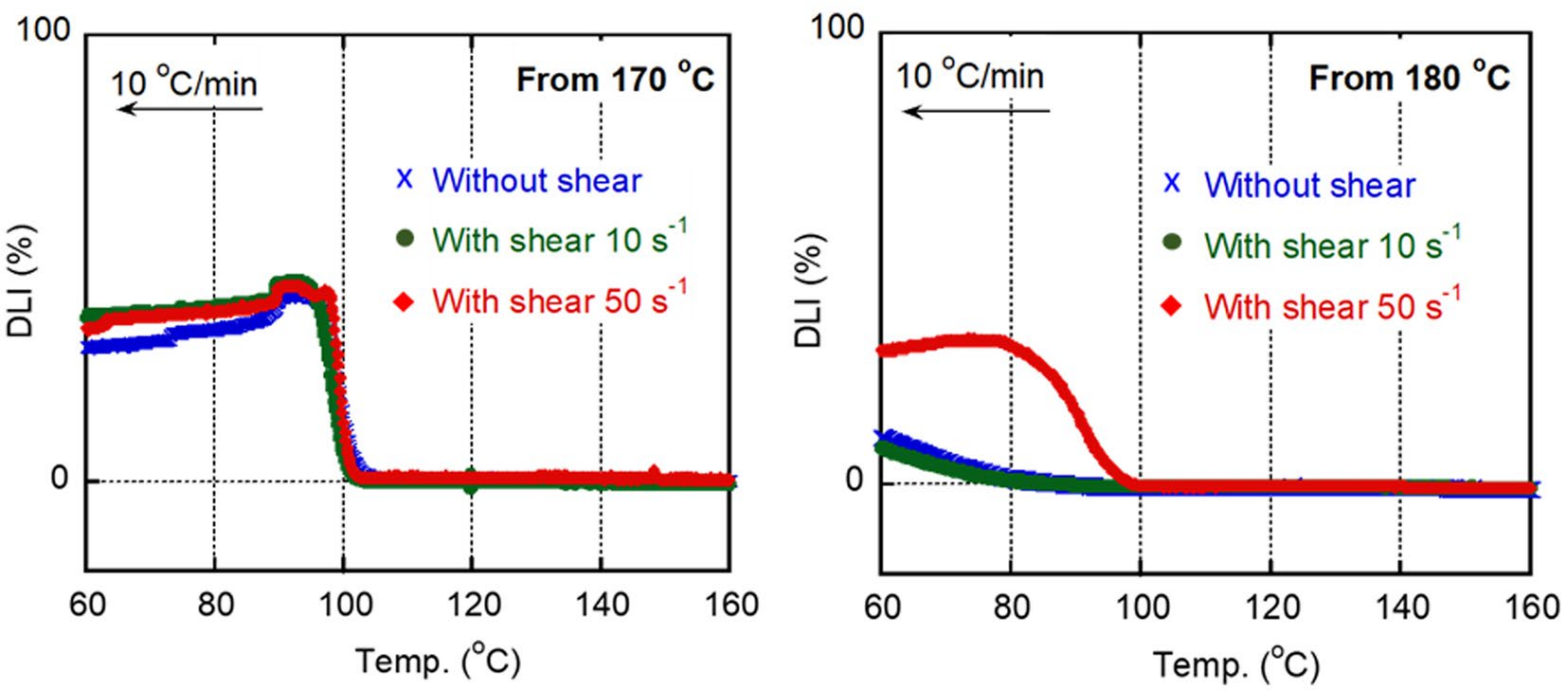
Growth curves of depolarized light intensity (DLI) with/without applied shear history during cooling at 10 °C min^−1^. The shear history was applied from 170 °C (left) and 180 °C (right) to 130 °C at 10 and 50 s^−1^.

Figure 6 shows the DLI curves obtained during cooling from 170 or 180 °C to 130 °C with shear history. When the sample was cooled from 170 °C, the shear history did not affect the crystallization temperature. The crystal residues must play a dominant role in crystallization. When cooled from 180 °C, in contrast, the crystallization occurred at a higher temperature after shear history was applied at 50 s^−1^, demonstrating that shear-induced crystallization occurred. The critical shear rate for the shish formation must be between 10 and 50 s^−1^.

**Figure 7:**
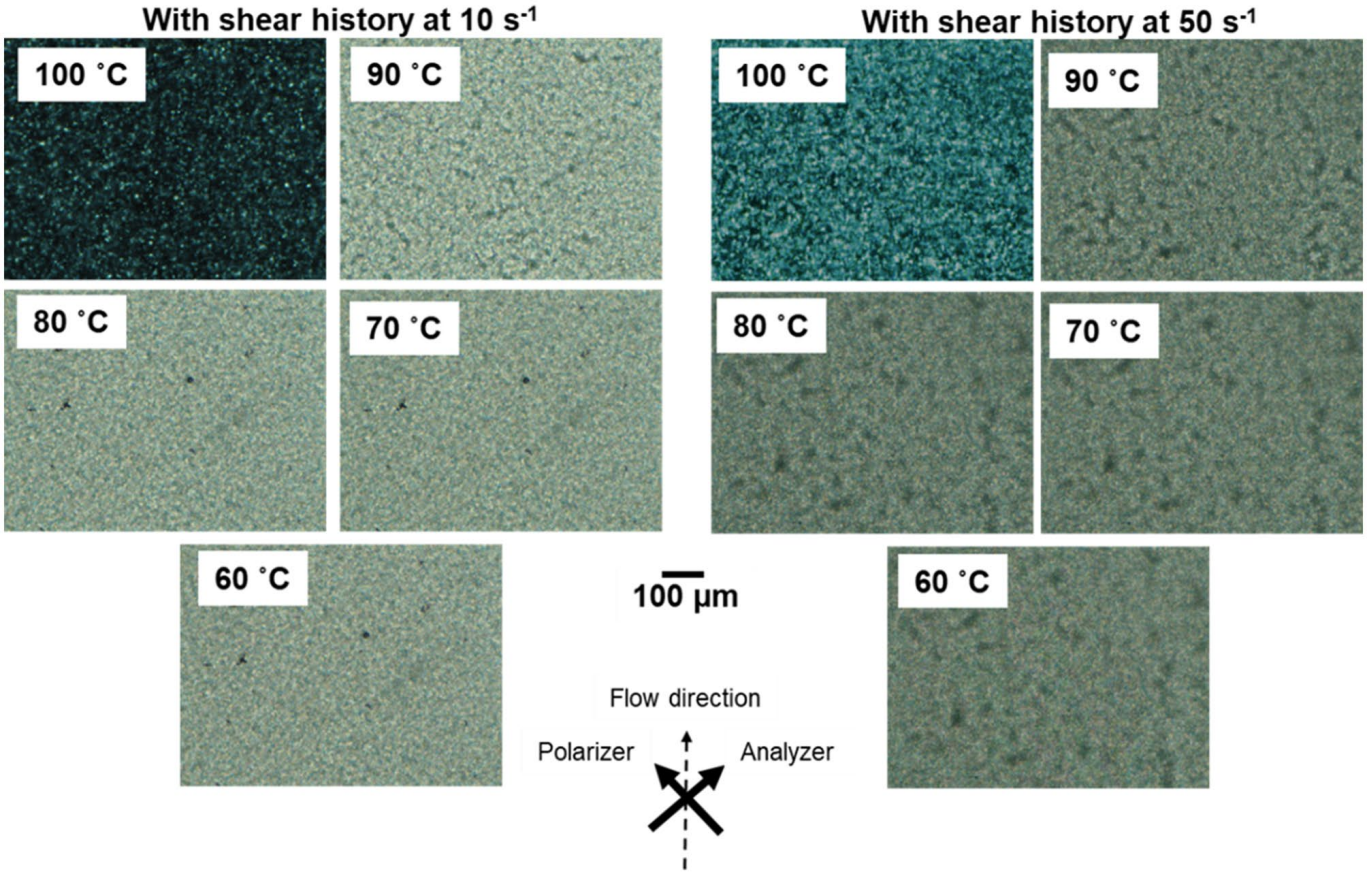
Polarizing optical microscope (POM) images showing the morphology of a sample. The images were obtained during cooling at shear rates of 10 and 50 s^−1^. The shear history was applied from 170 to 130 °C.

**Figure 8:**
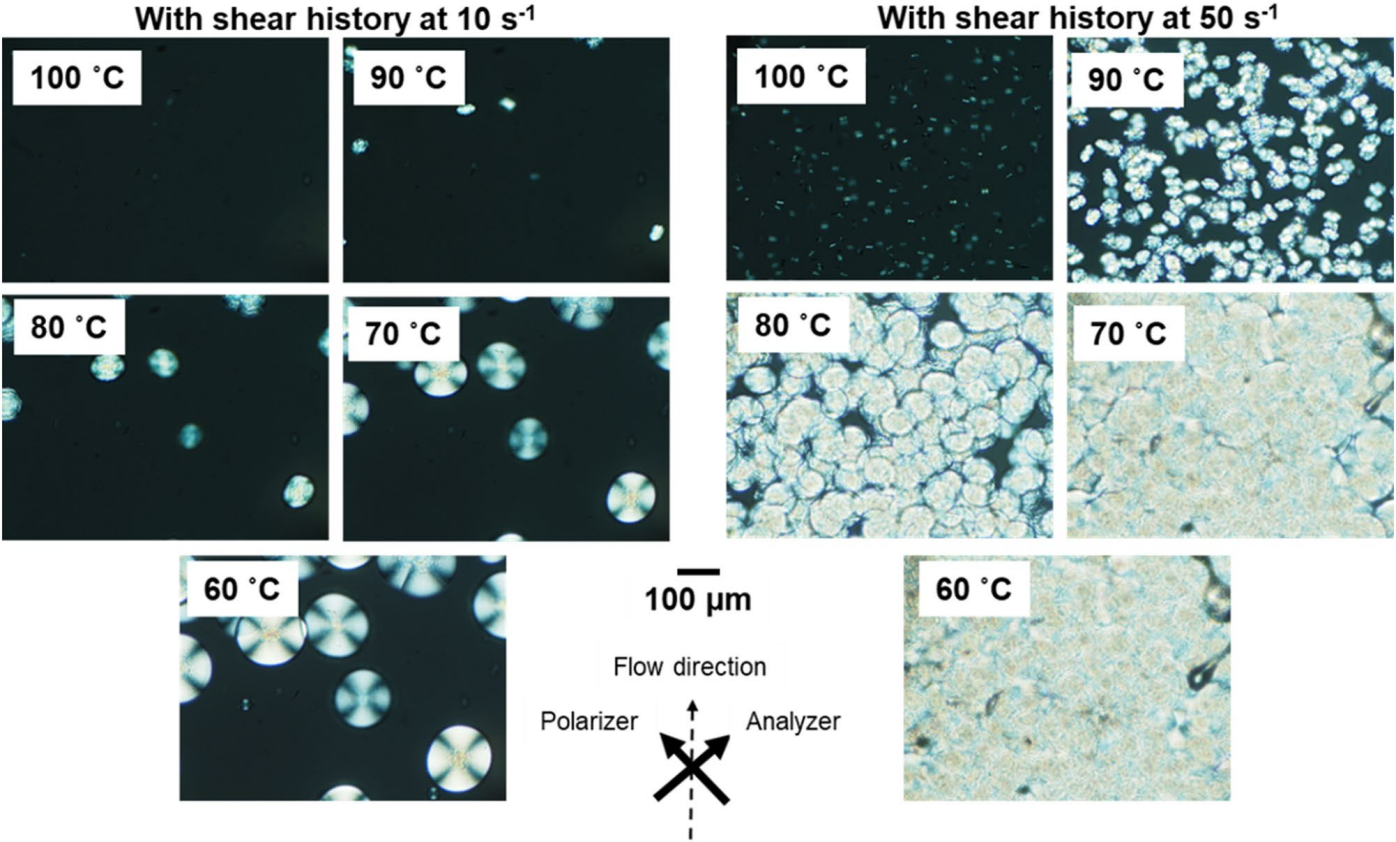
Polarizing optical microscope (POM) images showing the morphology of a sample. The images were obtained during cooling at shear rates of 10 and 50 s^−1^. The shear history was applied from 180 to 130 °C.

The POM images obtained with crossed polar configuration were shown in Figs. 7 and 8. The morphologies shown in Fig. 7 were almost similar to the top images in Fig. 5. This is as expected because the applied shear did not affect the crystallization kinetics. Crystallization occurred promptly owing to the presence of the unmolten crystals. The crystallization behavior was significantly different when the sample was cooled from 180 °C. As shown in Fig. 8, the morphologies after exposure to a shear of 10 s^−1^ were similar to those without shear history (Fig. 5, bottom). After shearing at 50 s^−1^, the number of spherulites increased markedly, as reported previously^10,18^. However, the applied shear flow did not affect the shape of the spherulites. The present result suggested that the chain orientation provided by the shear was fully relaxed before the lateral growth of the crystals.

**Figure 9:**
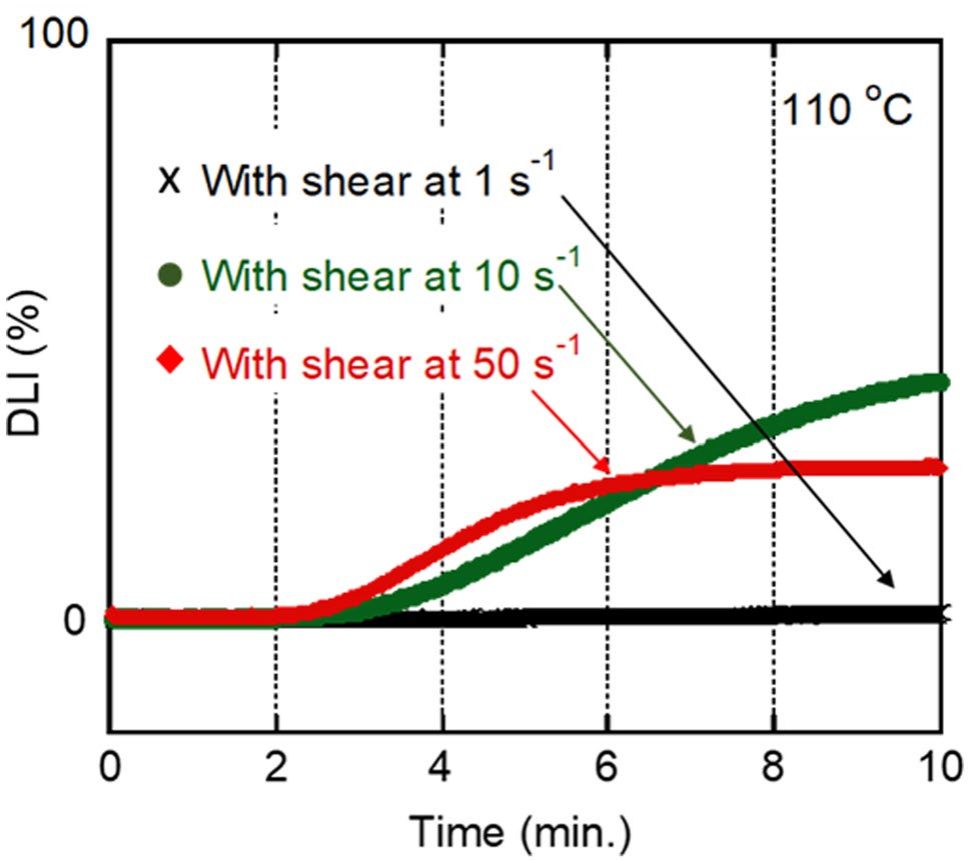
Growth curves of depolarized light intensity (DLI) during the isothermal process with applied shear history during cooling at 10 °C min^−1^. The shear history was applied from 180 to 110 °C at 1, 10, and 50 s^−1^, then held at 110 °C for 10 min.

**Figure 10:**
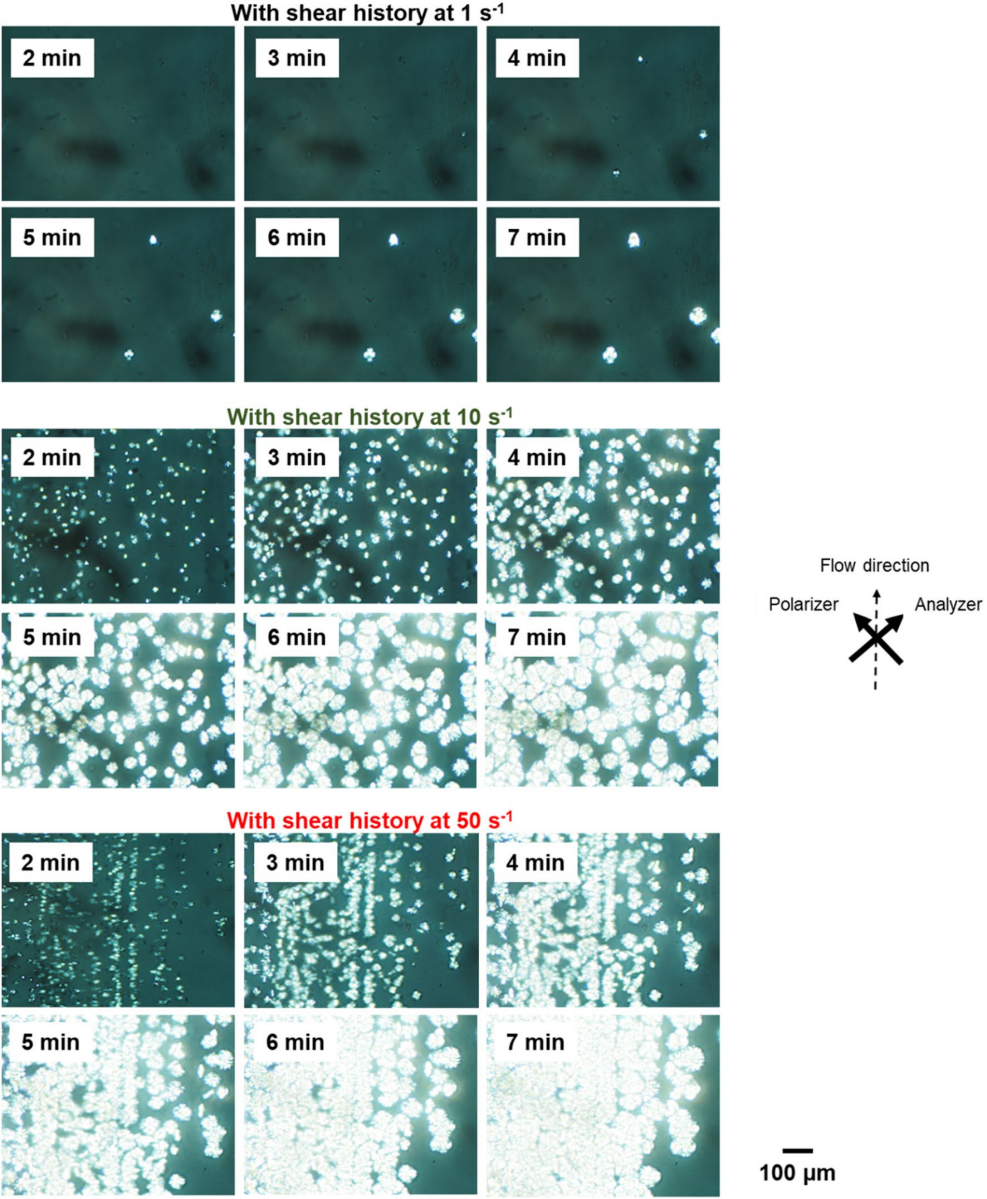
Polarizing optical microscope (POM) images showing the morphology during the isothermal process with applied shear history during cooling at 10 °C min^−1^. The shear history was applied from 180 to 110 °C at 1, 10, and 50 s^−1^, then held at 110 °C for 10 min.

Figures 9 and 10 show the isothermal crystallization behaviors at 110 °C. The samples were cooled at shear flows of 1, 10, and 50 s^−1^ to 110 °C, i.e., just before the isothermal process. The shear flow had a marked influence on the crystallization rate. In particular, shear flows of 10 and 50 s^−1^ greatly reduced the crystallization induction period; 180 s for 10 s^−1^ and 150 s for 50 s^−1^. Furthermore, as indicated in Fig. 10, the applied shear flow did not lead to shish-kebab structure but increased the number of crystal nuclei. Moreover, it should be noted that a number of spherulites were aligned in a row to the flow direction after the shear history at 50 s^−1^. This anomalous structure suggested the existence of extended chain crystals; i.e., shish was presumably formed during flow. However, it was not directly detected by investigation using the POM, because the diameter must be too small^30,32–35^. Subsequently, a part of the shish sporadically became a nucleus for spherulites. Because the shish oriented to the flow, i.e., vertical direction, spherulites aligned in vertical lines in the sample having the shear history at 50 s^−1^. The POM image also demonstrated that spherulites did not exhibit orientation/deformation to the flow direction, indicating that lateral growth at the spherulites occurred in all directions. This result demonstrated that even extended chain crystals cannot act as good nucleating agents to induce the transcrystallization for PHBHHx. Although the reason to form spherulite texture, not kebab, is unknown, there is a possibility that the crystalline form of shish is different from that of the folded chain crystals.

The weight-average relaxation time *τ*_*w*_ at 110 °C was predicted by the flow activation energy *E*_*a*_, and was found to be 0.040 s. Therefore, the Weissenberg number associated with reptation motion^33^, given by Eq. (9), became larger than 1 at 50 s^−1^. The result indicates the possibility of the anisotropic growth of crystals at the early stage of crystallization. However, because of the slow linear growth rate of PHBHHx, most molten chains lost their orientation before crystallization, which resulted in normal spherulites.

9 $$Wi_{rep} \equiv \tau_{rep} \dot{\gamma }.$$

Moreover, the Weissenberg number associated with the Rouse mode, defined in Eq. (10), must be larger than 1 at 10 s^−1^ because the number of crystals increased markedly.

10 $$Wi_{R} \equiv \tau_{R} \dot{\gamma }.$$

The Rouse relaxation time is determined by the molecular weight as follows:

11 $$\tau_{R} = \tau_{e} \left( {\frac{M}{{M_{e} }}} \right)^{2} ,$$

where *τ*_*e*_ is the Rouse relaxation time of a chain segment between neighboring entanglement points, and is reported to be 3 × 10^–4^ s for PHB^34^. Assuming that the *M*_*e*_ is 10,700, the molecular weight required for shish formation at $\dot{\gamma } = 10$ s^−1^ must be greater than 195,000. That is higher than the *M*_*z*+1_ of the present sample, which is 183,000 (the absolute molecular weight). This is as expected because a tiny amount of a high-molecular-weight fraction, e.g., 0.1%^46^, is sufficient for shish formation.

## Conclusions

The crystallization behavior of PHBHHx was studied considering actual processing conditions. The melt temperature before cooling markedly affects the crystallization rate. Heating a sample at/above 180 °C is not recommended because all the crystal residues melt, resulting in slow nucleation. Melting at 170 °C retained the unmolten crystals without affecting the rheological properties. These unmolten crystals act as nucleating agents and greatly increase the crystallization rate. The result indicated that unmolten crystals easily exist even without adding another PHBHHx having no/fewer hydroxyhexanoate content. This is important information to accelerate the crystallization rate especially at secondary processing operations, such as thermoforming, injection-blow-molding, and hot-stretching. When the unmolten crystals are melted by exposure to a high temperature, a strong shear flow should be applied, at which the Weissenberg number associated with Rouse mode is larger than unity. This situation promotes shish formation. However, an oriented crystal structure such as shish kebab is largely absent after the cessation of flow. In the present study, instead of the formation of an oriented crystal structure, several spherulites appeared in a row oriented with the flow direction. This unique structure must originate from the sporadic growth of spherulites on the extended chain crystals.

## Data Availability

All datasets used and/or analyses carried out and results obtained are available from the corresponding author on reasonable request.
